# ARR17 controls dioecy in *Populus* by repressing B-class MADS-box gene expression

**DOI:** 10.1098/rstb.2021.0217

**Published:** 2022-05-09

**Authors:** Ana P. Leite Montalvão, Birgit Kersten, Gihwan Kim, Matthias Fladung, Niels A. Müller

**Affiliations:** Thünen Institute of Forest Genetics, Sieker Landstrasse 2, 22927 Grosshansdorf, Germany

**Keywords:** cytokinin, dioecy, flower development, poplar, single gene sex determination

## Abstract

The number of dioecious species for which the genetic basis of sex determination has been resolved is rapidly increasing. Nevertheless, the molecular mechanisms downstream of the sex determinants remain largely elusive. Here, by RNA-sequencing early-flowering isogenic aspen (*Populus tremula*) lines differing exclusively for the sex switch gene *ARR17*, we show that a narrowly defined genetic network controls differential development of female and male flowers. Although *ARR17* encodes a type-A response regulator supposedly involved in cytokinin (CK) hormone signalling, clustered regularly interspaced short palindromic repeats (CRISPR)-Cas9-mediated *arr17* knockout only affected the expression of a strikingly small number of genes, indicating a specific role in the regulation of floral development rather than a generic function in hormone signalling. Notably, the *UNUSUAL FLORAL ORGANS* (*UFO*) gene, encoding an F-box protein acting as a transcriptional cofactor with *LEAFY* (*LFY*) to activate B-class MADS-box gene expression, and the B-class gene *PISTILLATA* (*PI*), necessary for male floral organ development, were strongly de-repressed in the *arr17* CRISPR mutants. Our data highlight a CK-independent role of the poplar response regulator ARR17 and further emphasize the minimal differences between female and male individuals.

This article is part of the theme issue ‘Sex determination and sex chromosome evolution in land plants’.

## 1. Introduction

Poplars are dioecious trees with a genetically controlled system of sex determination [1]. The genomic architecture of sex determination varies between species. The sex-determining regions (SDRs) have different locations and sizes [2–5], and different species exhibit different heterogametic systems [4–6]. Several studies have characterized the genetic basis of sex determination in poplars [2–5,7], including an experimental validation of a single-gene sex switch, named *ARR17*, in early-flowering aspens (*Populus tremula*) [4]. This gene likely underlies sex determination in both XY and ZW systems [4,5]. Although previous RNA-sequencing studies in different *Populus* species provided valuable insights into sexual development [8–10], the molecular function of ARR17 and the downstream regulatory pathways remained largely elusive. In particular, the possible involvement of cytokinin (CK) hormone signalling and the molecular pathways connecting ARR17 and B-class MADS-box gene expression represent open questions.

CK is a phytohormone that plays a crucial role in plant growth and development including sexual development, especially the gynoecium [11]. CK is perceived via a two-component system in which signal transduction is achieved by phosphorylation of response regulators (RRs) by histidine kinases (HKs), similar to the two-component systems employed by bacteria to respond to environmental stimuli [12,13]. The RRs are particularly interesting as they cause CK-dependent transcriptional reprogramming [12,14]. Since the poplar sex switch gene *ARR17* is homologous to the *ARABIDOSPIS RESPONSE REGULATOR 17*, one of the type-A RRs that are reported to negatively regulate the CK signalling cascade [15], a connection between *ARR17* and the CK pathway might be expected. Differential sexual development may be controlled by differential hormone signalling.

The separation of the sexes in dioecious species with type II flowers, that is flowers that are unisexual from inception [16], is thought to depend on proper temporal and spatial expression of floral homeotic genes [17,18]. According to the ABC model of floral development [19], floral organs (i.e. sepals, petals, stamens and carpels) are arranged in four distinct whorls, and within a regulatory network, the whorl-specific combination of homeotic gene expression determines floral organ identity. Different genes were found for each class encoding MADS-box transcription factors in *Arabidopsis thaliana* [20]. The A-class gene *APETALA 1* (*AP1*) is responsible for sepal development. The B-class genes *PISTILLATA* (*PI*) and *APETALA 3* (*AP3*) specify the petals and stamens depending on whether they are expressed together with A-class or C-class genes. Finally, the C-class gene *AGAMOUS* (*AG*) determines carpel development [18–20].

The B-class genes *PI* and *AP3*, which are essential for stamen development, have been highlighted as differentially expressed male-biased genes in different dioecious species, such as the persimmon *Diospyros lotus* [21] and the balsam poplar *Populus balsamifera* [9]. However, the molecular pathways connecting the sex switch genes *MeGI* in persimmon or *ARR17* in poplar with the floral MADS-box genes have remained unclear. In this study, we aimed to specifically characterize the molecular function of the poplar sex switch ARR17. To this end, we generated transcriptomic data of isogenic early-flowering male and female aspen lines only differing for a point mutation in the *ARR17* gene. These data allowed us to investigate the molecular mechanisms downstream of ARR17 without the confounding effects of different genetic backgrounds. We find that, in poplar, ARR17 functions independently of CK and triggers female development by repressing the *UNUSUAL FLORAL ORGANS (UFO)*–*PI* cascade, suggesting a direct role of ARR17 on the specification of floral organ identity.

## 2. Material and methods

### (a) Plant material, growth and sampling

The plant material (flower buds) was obtained from one female early-flowering line (T222-3), which expresses the *A. thaliana FLOWERING LOCUS T* (*FT*) gene under the control of the heat-inducible promoter derived from the soya bean gene *hs6871* [22] encoding a heat shock protein (HSP), and three independent T222-3-based isogenic *arr17* CRISPR mutants (N500-1, N500-3 and N500-5) previously described [4]. Each of these lines contains a clustered regularly interspaced short palindromic repeats (CRISPR)-induced mutation disrupting the open reading frame of *ARR17*. *In vitro*-grown plants were transferred to soil and cultivated under 16/8 h light/dark and 22/17°C temperature cycles for 1.5 months. To induce *FT* expression and the consequential development of generative buds, a heat shock treatment was applied for 2 h at 40°C every day for one month. The plants were randomized and watered daily. The experiments were conducted in two batches, under the same conditions. For the first batch, the flower buds were sampled every 5 days after the start of the heat shock treatment until fully formed flowers were observed. Samples from days 5, 10, 15 and 20 were used for RNA-sequencing. For each time point, three biological replicates were collected for each sex (female: 3× T222-3, *arr17* CRISPR: 1× N500-1, 1× N500-3 and 1× N500-5). Each replicate consisted of flower buds pooled from three plants. The second batch was prepared the same way; however, only samples from day 20 were used for RNA-sequencing. For *Populus alba*, flower buds from a female (clone Jap1) and a male (clone Monrepos) field-grown tree (three samples per tree) were collected at a single time point on 22 July 2020. All flower buds were snap frozen in liquid nitrogen and stored at −70°C until RNA extraction.

### (b) RNA extraction, cDNA synthesis and qRT-PCR chain reaction

The frozen flower buds were ground to a fine powder in a Retsch mill (Retsch GmbH, Germany) at 25 Hz for 30 s and this powder was used for RNA extraction. Total RNA was extracted with the Spectrum Plant Total RNA kit (Sigma-Aldrich, USA) according to the manufacturer's manual, Protocol A. Following that, DNase I digestion was performed using the Turbo DNA-free kit (Invitrogen, USA). The RNA concentration and purity were assessed using a Nanodrop 1000 spectrophotometer (Peqlab Biotechnologie GmbH, Germany) and by native agarose gels. The RNA Integrity Number (RIN) was determined using the plant-specific protocol of Agilent Bioanalyzer (Agilent Technologies, Inc., USA). All samples presented RIN greater than 7. For complementary DNA (cDNA) synthesis, 2 µg of RNA, Oligo (dT) primers and SuperScript IV reverse transcriptase (Invitrogen, USA) were used following the manufacturer's protocol, using 10 µl reactions without RNaseOUT. Reverse transcriptase quantitative polymerase chain reaction (qRT-PCR) was carried out in duplicates on a CFX96 Touch Real Time PCR Detection System (Bio-Rad Laboratories GmbH, USA) using the SsoAdvanced Universal SYBR Green Supermix (Bio-Rad Industries, Inc., USA) and a two-step PCR programme with annealing temperature of 60°C. Relative expression levels were calculated using the 2^−ΔΔ^Ct method [23]. The primers are given in electronic supplementary material, table S1.

### (c) RNA-sequencing and data analysis

Strand-specific RNA-seq libraries were generated by Novogene (Novogene (UK) Company Ltd., Cambridge, UK) and sequenced using the Illumina HiSeq platform. Paired-end 150 bp reads to a target depth of 30 million paired-end reads per sample were produced. The filtering of sequenced reads consisted of removing reads containing adapters, reads containing undetermined bases (*N* > 10%) and low-quality reads (*Qscore* ≤ 5). The quality of the raw reads was assessed using FastQC [24]. The reads were mapped to the *P*. *tremula* v. 2.2 genome [25] using STAR aligner (v. 2.7.1a) with default settings and with the annotation gene file, and they were subsequently used to calculate read counts with the R package Rsubread [26] and the command *featurecounts*. Differential expression (DE) analyses between the lines was performed in R v. 4.0.4 using the DESeq2 package (v. 1.30.1) [27]. The raw dataset was filtered by removing genes for which the sum of reads for all samples was below 10. From the second experiment, an outlier sample was removed from further analysis, since no reads of *ARR17* were detected (electronic supplementary material, figure S1). The remaining samples and genes were used for the DE analysis using *DESeq* function (design = ∼batch + sex). The adjusted *p*-value (*p*_adj_ < 0.05) and an absolute Log_2_FoldChange (log_2_FC) greater than 1.5 were used to assess significance and identify differentially expressed genes (DEGs). The variance-stabilizing *rlog* was used, and the counts were normalized using DESeq2's own normalization method for exploratory analyses such as principal component analysis (PCA). Batch effects were removed using the *removeBatchEffect* function from the R package limma (v. 3.46.0) [28]. The raw read counts are given in electronic supplementary material, table S2.

### (d) Gene set enrichment analysis based on gene ontology

A GO term enrichment analysis was performed using the topGO package in R (v. 2.42.0) [29] with default settings as well as the optional function *nodeSize* = 10, which removes terms with fewer than 10 annotated genes. We considered 24 464 genes (out of the 29 549 expressed genes used for the DE analyses) that had a GO annotation for *P*. *tremula* [25]. The analysis was performed with DEGs at our standard cut-off (*p* < 0.05 and |log_2_FC| > 1.5) and using a relaxed significance level of *p* < 0.1 to avoid false negatives and to control for random effects due to threshold choice [30].

### (e) Cytokinin treatment

Female and male heat-inducible early-flowering aspen lines were grown for one month in tissue culture and subsequently transferred to Magenta plant incubation boxes (Sigma-Aldrich, USA) containing woody plant medium (WPM) with and without 6-benzylaminopurine (BAP), which is a synthetic CK that promotes growth and is involved in various developmental processes such as cell division, shoot formation and promotion of flowering. Different concentrations of BAP were used: 110, 220, 440 and 880 µM, in four individuals per sex, totalling 32 treated plants. Moreover, six plants of each sex were used as a control (without BAP). Following a three-week incubation in a climate room at 21°C and constant light, the cultures were placed under a daily heat treatment (2 h at 40°C) to trigger flowering as described above. Flowers were analysed after 45 days, and the numbers were recorded.

## 3. Results and discussion

Expression of *ARR17* in poplar is tissue-specific, occurring only in female flower buds [4,31]. Thus, to identify the downstream genes controlled by *ARR17*, we analysed transcriptome datasets of female and male flower buds collected at different times of development. For reliable sample collection, we took advantage of an artificial flower induction system, which uses the *A. thaliana FT* gene under the control of a soya bean HSP promoter. This system enables heat-inducible early-flowering and allows poplar flower development—which in nature takes almost 1 year and is inconsistent between individuals and years [32,33]—to be compressed into one month by heat shock-mediated induction of *FT* expression [4,32]. During this month, we repeatedly sampled the developing flower buds of female and male early-flowering aspen (*P. tremula*) lines. Importantly, these female and male lines (henceforth referred to as female and *arr17* CRISPR) are genetically identical, except for a CRISPR-Cas9-induced *arr17* mutation. This single-induced mutation, which disrupts the open reading frame of *ARR17*, changes females to males [4]. These isogenic lines provide a unique possibility to study the genetic networks downstream of the sex-determining gene without any confounding effects from different genetic backgrounds, which usually complicate comparisons between female and male individuals.

**Figure 1.**
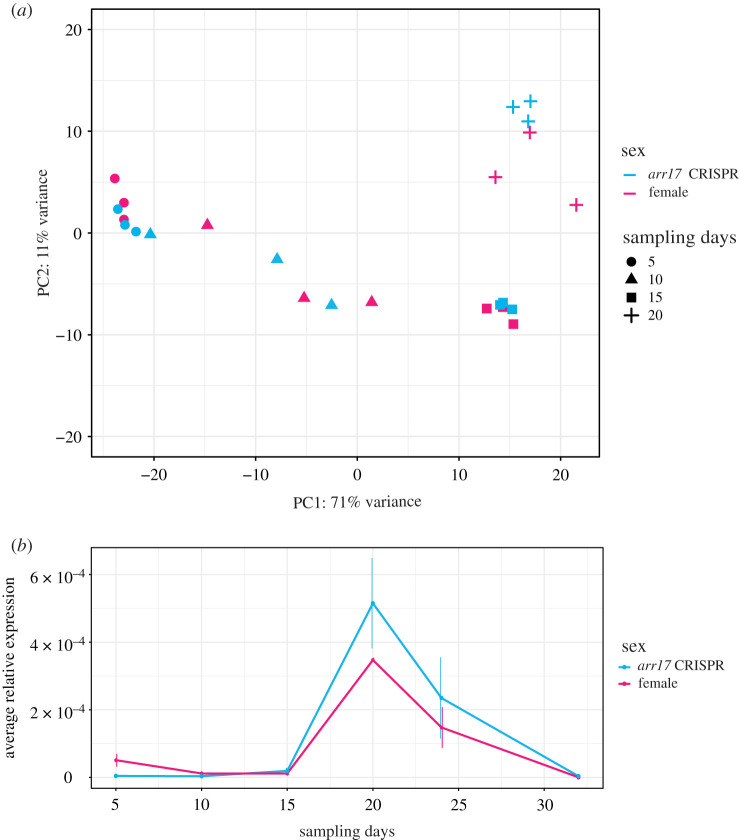
Transcriptome variation and *ARR17* expression in female and male flower buds of early-flowering aspen lines. (*a*) Principal component analysis (PCA) of transcriptome variation of early-flowering female (magenta) and male *arr17* CRISPR (blue) aspen (*P. tremula*) lines. Different symbols indicate different sampling days (i.e. days 5, 10, 15 and 20 after the start of flower induction). The first principal component PC1 explains 71% and the second principal component PC2 11% of the total variance. (*b*) *ARR17* expression occurs in a narrow temporal window during poplar flower development. Average relative expression (*n* = 3) of *ARR17* expression determined via qRT-PCR over a developmental time course in the same two genotypes shown in (*a*). *ARR17* expression peaks at day 20. Error bars indicate the standard error of the mean (SEM).

To assess DEGs downstream of *ARR17*, samples from days 5, 10, 15 and 20 after the start of flower induction were employed for RNA-sequencing, with three replicates per sex and day. Initially, we analysed the general patterns of transcriptome variation with a PCA (figure 1*a*). The PCA indicated rapid and dynamic changes in the transcriptome consistent with the transitioning from vegetative to generative development. PC1 and PC2 represented 71% and 11% of the total variance, respectively, and most of the variation in the transcriptome can be accounted for by the sampling day. Nevertheless, a marked separation between female and *arr17* CRISPR lines occurred at day 20 (figure 1*a*), suggesting that this may be the earliest stage of sexual differentiation. In line with this, day 20 was the first time point with robust *ARR17* expression in a qRT-PCR expression time course (figure 1*b*). *ARR17* expression was present in a narrow temporal window during the developmental trajectory from vegetative buds to fully developed flowers. These results highlight that *ARR17* expression is not only tissue-specific but also time-specific. Considering that ARR17 represents a single-gene sex switch, differences between females and males could hardly be smaller.

While *ARR17* expression is female-specific in the natural sex-determining systems [4,9], we see expression at day 20 also in our male *arr17* CRISPR lines (figure 1*b*). This can, however, be explained by the fact that the *arr17* CRISPR lines present a male phenotype due to a loss of function mutation of the *ARR17* gene at the protein level and not due to transcriptional silencing or gene absence. The peak of *ARR17* expression corresponds to an early stage of flower development (electronic supplementary material, figure S2a). Notably, no sex-specific differences in flower bud morphology or any other floral structures could be observed between female and *arr17* CRISPR lines at time point 20 (electronic supplementary material, figure S2b). A study in *Populus* *balsamifera* demonstrated that in nature the highest expression of *ARR17* also occurs at the earliest stages of reproductive development [9]. Together, these data suggest that *ARR17* may determine sex early on during flower development.

Since our developmental RNA-seq and *ARR17* qRT-PCR time courses demonstrated that the earliest substantial sex-specific differences are expected for day 20, we focused further differential gene expression analyses on that time point. It must be noted, however, that three biological replicates limit the statistical power to identify sex-specific differences. We only found two significantly (*p* < 0.05, |log_2_FC| > 1.5) DEGs, i.e. Potra2n2c5701 (*PI*) and Potra2n4c8755 (*BGLU13*) (electronic supplementary material, figure S3). We therefore generated a second identical RNA-seq dataset for day 20. The combination of replicates from both experiments should provide sufficient statistical power and allow the reliable identification of DEGs. A total of 29 549 expressed genes were analysed for DE (electronic supplementary material, table S3). To get a first overview of the biological processes that may be involved in sex determination, we performed a gene ontology (GO) term enrichment analysis. For this analysis, we selected DEGs based on different significance thresholds (using a relaxed setting: *p* < 0.1, and a more stringent setting: *p* < 0.05 and |log_2_FC| > 1.5), since the threshold choice can have a relevant effect on the results [30]. With both settings, we identified the biological processes ‘positive regulation of transcription by polymerase II' (GO:0045944) and ‘maintenance of meristem identity' (GO:0010074) among the top five categories (electronic supplementary material, tables S4 and S5). Strikingly, almost 90% (15/17) of the DEGs (*p* < 0.1) involved in positive regulation of transcription were MIKC-type MADS-box genes, which play prominent roles in the control of reproductive development [34,35]. In particular, all *AP3* and *PI* paralogues, which are essential for stamen development, were upregulated in the *arr17* CRISPR mutants (electronic supplementary material, table S5). The three identified meristem identity genes included both *UFO* paralogues, which encode F-box proteins acting as transcriptional cofactor with *LEAFY* (*LFY*) to activate B-class MADS-box gene expression and are reported to provide the spatial cues for the expression of *AP3* and *PI* [36,37]. No category related to the CK signalling pathway, such as ‘response to CK’ (GO:0009735), was enriched. These results argue against a function of ARR17 in modulating CK signalling to control sex determination in poplar, but rather highlight the importance of ARR17 in repressing MIKC-type MADS-box transcription factors to specify floral organ identity.

**Figure 2.**
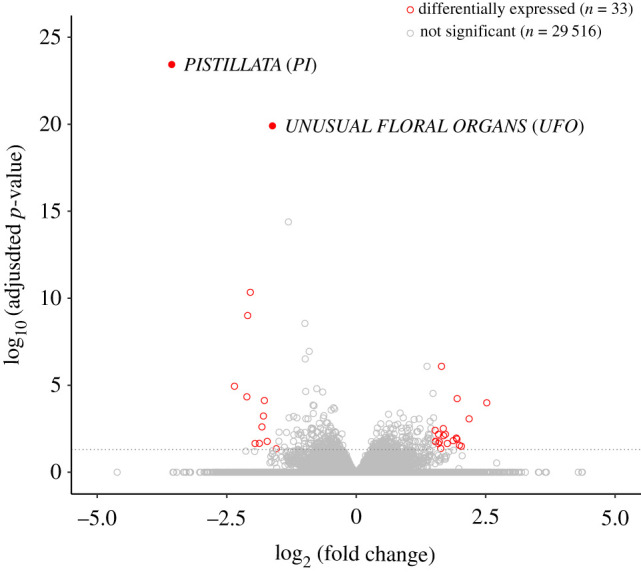
*PISTILLATA* (*PI*) and *UNUSUAL FLORAL ORGANS* (*UFO*) are strongly upregulated in *arr17* CRISPR mutants on day 20. Volcano plot showing 29 549 expressed genes. Significantly differentially expressed genes (female versus *arr17* CRISPR lines) at day 20 (*p*_adj_ < 0.05 and abs(log_2_FC > 1.5)) are depicted in red. *PI* and *UFO* are highlighted by filled symbols. The dashed grey line indicates the *p*-value significance threshold.

The differential gene expression analysis of the combined dataset with a standard significance cut-off (i.e. *p* < 0.05 and |log2FC| > 1.5) resulted in a strikingly small set of 33 DEGs (table 1 and figure 2), indicating minimal changes in the transcriptome upon *arr17* knockout. Among those 33 DEGs, 13 are upregulated in the *arr17* CRISPR mutants. Two of these genes stand out compared to all others: *PISTILLATA* (Potra2n2c5701) required for stamen development [38] and *UFO* (Potra2n1c1412), which activates B-class MADS-box gene expression [39–42]. Both these genes are strongly upregulated in the *arr17* CRISPR mutants (figure 2). It should be noted that their paralogues are also differentially expressed (table 1, rows 6 and 7). We were wondering whether the same genes may be differentially expressed in poplar species with independently evolved systems of sex determination. For example, *P. alba* features a ZW system of sex determination in which *ARR17* is located in the female-specific region of the W chromosome [4,5]. *Populus balsamifera* exhibits an XY system similar to the one found in the aspens but with a different genomic architecture and an independent evolutionary origin [2,4,43]. For *P. balsamifera*, 854 DEGs in early developing female and male floral buds (July 2017) have been reported before [9]. For *P. alba*, we generated RNA-seq data to assess differential gene expression (*p* < 0.05 and |log_2_FC| > 1.5) in female and male samples collected at an early stage of reproductive development (July 2020) as well. These data identified a total of 1725 DEGs (electronic supplementary material, table S6). All three datasets, which are not expected to share any gene by chance, shared exactly two DEGs representing the two *PI* paralogues (electronic supplementary material, figure S4 and table S7). *UFO* was not assessed in *P. balsamifera* but was shared between *P. tremula* and *P. alba* (electronic supplementary material, table S7). These additional data further highlight the prominent role of *PI* and *UFO* and suggest that the molecular mechanism of sex determination may be shared between species with independently evolved SDRs.

**Table 1.** Differentially expressed genes (DEGs) at day 20. For each gene, the Log_2_FoldChange (log_2_FC; female versus *arr17* CRISPR), the adjusted *p*-values (*p*_adj_), the respective *P*. *trichocarpa* and *A. thaliana* gene identifiers and the *A. thaliana* synonym are given.

Potra_v2.2_ID	log_2_FC	*p*_adj_	*P. trichocarpa* ID	*A. thaliana* ID	*A. thaliana* synonym
Potra2n2c5701	−3.56	1.99 × 10^−24^	Potri.002G079000	AT5G20240	pistillata (PI)
Potra2n1c1412	−1.62	1.01 × 10^−20^	Potri.001G160900	AT1G30950	unusual floral organs (UFO)
Potra2n10c20292	−2.05	3.17 × 10^−11^	Potri.010G236300	AT3G21510	histidine-containing phosphotransmiter 1 (AHP1)
Potra2n2c4152	−2.10	7.15 × 10^−10^	Potri.002G250000	AT3G25400	dCTP pyrophosphatase
Potra2n4c8755	1.65	7.41 × 10^−7^	Potri.004G040700	AT5G44640	beta glucosidase 13 (BLU13)
Potra2n3c7869	−2.35	8.11 × 10^−6^	Potri.003G074100	AT1G30950	unusual floral organs (UFO)
Potra2n5c11227	−2.11	3.66 × 10^−5^	Potri.005G182200	AT5G20240	pistillata (PI)
Potra2n16c29771	1.95	4.83 × 10^−5^	Potri.016G058500	AT4G38180	far1-related sequence (FRS5)
Potra2n2c5611	−1.77	6.07 × 10^−5^	Potri.002G088200	AT1G37140	MEI2- C-terminal RRM only like 1
Potra2n18c32797	2.52	8.14 × 10^−5^	Potri.018G053600	AT5G56860	GATA transcription factor 21 (GATA21/GNC)
Potra2n9c19634	−1.79	0.000513	Potri.009G055700	AT5G13790	agamous-like 15 (AGL15)
Potra2n10c21177	2.18	0.000764	Potri.010G141000	AT5G49330	MYB domain protein 111 (MYB111)
Potra2n299s35250	−1.82	0.002193	Potri.008G131100	AT1G70890	major latex protein-like 43 (MLP43)
Potra2n1c1097	1.68	0.002836	Potri.003G106800	AT5G51330	switch 1 (SWI1)
Potra2n6c13866	1.52	0.003592	Potri.006G165900	AT4G30190	plasma membrane protein ATPase 2 (PMA2)
Potra2n15c28326	1.72	0.006144	Potri.005G036600	AT1G54820	protein kinase superfamily protein
Potra2n5c12526	1.59	0.006144	Potri.015G095900	AT5G50400	purple acid phosphatase 27 (PAP27)
Potra2n2c5221	1.69	0.007187	Potri.014G038500	—	
Potra2n11c23459	1.93	0.010079	Potri.011G031800	AT3G25820	terpene synthase-related protein (TPS-CIN)
Potra2n5c12753	1.95	0.011358	Potri.005G014900	AT4G21390	S-locus lectin protein kinase family protein (B120)
Potra2n6c13588	1.87	0.014159	Potri.006G199300	AT1G68450	pigment defective 337 (PDE337)
Potra2n5c12584	1.53	0.015329	Potri.005G028200	AT3G26040	HXXXD-type acyl transferase
Potra2n2c4059	−1.72	0.016211	Potri.014G195800	AT5G44070	phytochelatin synthase 1 (PCS1)
Potra2n6c14378	1.53	0.016528	Potri.006G107700	AT2G30400	ovate family protein 2 (OFP2)
Potra2n10c20471	1.61	0.016988	Potri.008G043900	AT1G07900	LOB domain-containing protein 1
Potra2n12c23975	−1.87	0.020274	Potri.012G032300	AT5G15290	domain of unknown function (DUF588)
Potra2n13c25563	−1.95	0.021018	Potri.013G084400	AT3G26120	terminal ear1-like (TEL1)
Potra2n432s35661	1.76	0.021045	Potri.001G015400	AT3G45140	lipoxygenase 2 (LOX2)
Potra2n1c775	1.58	0.021279	Potri.003G138400	AT5G42800	dihydroflavonol 4 reductase (DFR4)
Potra2n6c15208	1.99	0.026446	Potri.006G019800	—	
Potra2n3c7698	2.03	0.029643	Potri.003G091200	AT4G17810	zinc finger protein 1 (ZP1)
Potra2n18c32253	1.63	0.04113	Potri.018G113300	AT4G02050	sugar transporter protein 7 (STP7)
Potra2n14c27869	−1.54	0.041274	Potri.014G179400	AT1G32450	nitrate transporter 1.5 (NRT1.5)

Interestingly, key genes from the CK signalling pathway such as the type-B RRs *ARR1*, *ARR10* and *ARR12* are not differentially expressed, indicating that ARR17 may not be involved in CK signalling (electronic supplementary material, figure S5). This is in line with the GO term enrichment analyses, which also failed to detect any connection of *arr17* mutation with CK signalling. CK-independent roles of type-A RRs have been described before. For instance, in *A. thaliana*, the type-A RRs ARR3 and ARR4 play CK-independent roles in the circadian clock [44]. To further explore the potential role of CK signalling in poplar sex determination, we adapted our early-flowering system to generate poplar plants flowering *in vitro* with a height of only 10 cm. This system allowed us to test the effect of exogenous application of synthetic CK into the growth medium on flower development. In particular, we wanted to assess whether treatment with 6-BAP may have any effect on sexual development. While we observed an increase in the total number of flowers (electronic supplementary material, figure S6), there was no effect on flower sex. This is in contrast with other dioecious species where an exogenous application of CK in male flowers stimulates the induction of carpel development, converting them to hermaphrodites [45–47].

**Figure 3.**
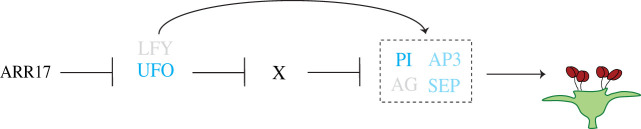
Potential downstream pathway of the sex switch ARR17. For the development of male flower organs (i.e. stamens), the B-class MADS-box genes *PISTILLATA* (*PI*) and *APETALA 3* (*AP3*) are essential as they form a heterodimer (dashed square) with C-class and E-class genes *AGAMOUS* (*AG*) and *SEPALLATA* (*SEP*), respectively. These genes are regulated transcriptionally by genes such as *LEAFY* (*LFY*) and the cofactor *UNUSUAL FLORAL ORGANS* (*UFO*) (as part of the SCF complex), which potentially represses a factor (depicted as ‘X') via degradation [50] that would repress B-class gene expression. (Online version in colour.)

In summary, our results suggest a specific function of the poplar sex switch gene *ARR17* on floral organ identity rather than a generic function in the CK signalling pathway. The poplar *ARR17* gene is orthologous to the *A. thaliana* gene pair *ARR16*/*ARR17*. There is no one-to-one orthology. In *A. thaliana*, overexpression of the *ARR16* and *ARR17* genes slightly affects flowering time but does not appear to change floral organ identity [48]. Additionally, the *ARR16*/*ARR17* gene pair appears to be specifically involved in regulating cell divisions of the stomatal lineage [49]. By contrast, our results in poplar highlight *UFO* and *PI* as major downstream factors and thus the regulation of floral organ identity as the key function of the sex determinant ARR17. In the absence of ARR17 activity, expression of *UFO* is ensured, and as a transcriptional cofactor with *LFY*, it activates B-class MADS-box genes [40,50]. On the other hand, in females, ARR17 prevents the expression of *UFO* and therefore represses male development (figure 3).

Remaining open questions concern the mechanism by which ARR17 prevents *UFO* expression in females, the linearity of the pathway and the presence of possible additional factors on the male Y chromosome that might contribute to differential sexual development in nature. ARR17 is a single-domain RR because it contains only a receiver domain [51]. The C-terminal extension in ARR17 of *P. tremula* is short (only nine amino acids in Potra000483g02981.1; electronic supplementary material, figure S7), similar to ARR16 and ARR17 in *A. thaliana* [52]. The absence of any kind of effector domain at the C-terminus in ARR17 argues against direct transcriptional control of *UFO* by ARR17. Single-domain RRs may rely on protein–protein interactions to exert their downstream biological effects, after phosphorylation by a HK and conformational change of the receiver domain [53]. Thus, one mode of action could be that ARR17 interacts at the protein level with a transcriptional regulator of *UFO*. Regarding the linearity of the pathway, ARR17 could be involved in the repression of *UFO* and *PI* only, or it could fulfil additional essential functions to determine the sex of poplars. This question should be addressed in future experiments by knocking out the poplar genes *UFO* and *PI*. In the case of a linear pathway, *ufo* and *pi* mutations should convert males to females.

## 4. Conclusion

RNA-sequencing of developing flower buds of early-flowering isogenic female and male aspen lines only differing for a CRISPR-induced mutation in the sex determinant *ARR17* identified DEGs likely involved in poplar sex determination. During poplar development*, ARR17* is only expressed in floral buds and only in a narrow temporal window during flower bud development. The difference between females and males could hardly be smaller, which is in line with sexual homomorphism reported in different poplar species [7,54,55]. Despite being a type-A RR, ARR17 does not appear to control CK signalling. Instead, *UFO* and the B-class MADS-box gene *PI* were highlighted by several analyses as key components of the gene network downstream of ARR17 (figure 3), indicating a highly targeted role of ARR17 in specifying floral organ identity. It will be exciting to explore the proposed pathway further and to generate *ufo* and *pi* knockouts to test whether the modulation of additional signalling cascades is essential or whether the repression of *UFO* and *PI* alone is sufficient to specify differential sex expression.

## Data Availability

The RNA-sequencing data were deposited in NCBI's SRA under the bioproject accession number PRJNA773612.
